# Horticultural therapy for stress reduction: A systematic review and meta-analysis

**DOI:** 10.3389/fpsyg.2023.1086121

**Published:** 2023-07-26

**Authors:** Shan Lu, Jianjiao Liu, Meijing Xu, Feng Xu

**Affiliations:** ^1^Department of Landscape Architecture, College of Horticulture, China Agricultural University, Beijing, China; ^2^Faculty of Architecture, Building and Planning, Melbourne School of Design, University of Melbourne, Parkville, VIC, Australia

**Keywords:** horticultural activities, stress, environmental settings, theoretical framework, meta-analysis

## Abstract

**Introduction:**

Horticultural therapy has been increasingly accepted as a non-pharmacological stress reduction treatment. Previous studies have demonstrated its therapeutic effects, with the effect varying according to the populations, settings, and interventions of horticultural therapy. This study aimed to provide a comprehensive review of the current literature regarding the effectiveness of horticultural therapy in reducing stress.

**Methods:**

We selected databases including PubMed, Cochrane Library, Embase, Web of Science, China National Knowledge Infrastructure, and VIP Data as our data source, and the original search was completed in January 2023.

**Results:**

Our results showed significantly increased effects of horticultural therapy on psychological indicators compared to a control group, but an insignificant effect on physiology indicators. The result of the subgroup analysis demonstrated that the stress-reducing effects of horticultural therapy were related to the characteristics of the population and indoor and virtual areas were the most effective setting for horticultural therapy. At the same time, a total duration of 100–500 minutes provided better effects of stress reduction.

**Discussion:**

We also developed a theoretical framework based on a “Participants-Settings-Interventions” structure for horticulture therapy in terms of its stress-reduction effects, to provide a reference for future horticultural therapy activities.

## 1. Introduction

With the ongoing trend of urbanization, more than two-thirds of the world's population is expected to live in cities and towns by 2050 (Montgomery, 2007). In the same time frame, there is an increasing number of people suffering from stress-related issues (Dye, 2008). In fact, stress-related mental health issues such as depression and anxiety will become more prevalent by 2030, according to the World Health Organization (World Health Assembly, 2012). Individual stress can ultimately reduce the productivity and general wellbeing of society as a whole (Vinokur and Caplan, 1986), at the same time increasing the burden on the government's investment in public health (Greenberg et al., 1999; Ho et al., 2013).

Stress-related issues have always been a major focus of medical and psychological research. There are many stress-inducing factors, including an actual or perceived threat to an organism, which is referred to as the “stressor” (Schneiderman et al., 2005). Stressors typically include personal difficulties (e.g., conflict with loved ones, being alone, lack of income, worries about the future), problems at work (e.g., conflict with colleagues, an extremely demanding or insecure job), or major threats in the community (e.g., violence, disease, lack of economic opportunity) (World Health Organization, 2020). The response to stressors is known as “stress response”, an adaptive mobilization of the organism to cope with potentially negative situations (Kaplan, 1995) and any effects that seriously threaten homeostasis (Selye, 1978). It could be linked to vascular (Katsarou et al., 2013), neurological (Busciglio et al., 1998), autoimmune (Stojanovich and Marisavljevich, 2008), cardiovascular (Esch et al., 2002; Pogosova, 2007), inflammatory illness (LeResche and Dworkin, 2002), and other disorders, and might lead to the aggravation of diabetes (Wellen and Hotamisligil, 2005) and asthma (Ohno, 2017). The unprecedented stress caused by social isolation from the COVID-19 pandemic has been proven to lead to anxiety and depression (Santomauro et al., 2021). Therefore, there is an urgent need for appropriate methods to address stress-related problems.

Horticultural therapy has been increasingly embraced as a non-pharmacological stress reduction treatment due to its flexibility and free of side effects. Horticultural therapy encourages people to spend time in nature, which has been shown to have stress-relieving and attention-restoring effects, based on the Stress Recovery Theory (SRT) (Ulrich et al., 1991) and the Attention Restoration Theory (ART) (Kaplan, 1995). In recent decades, researchers and health practitioners have placed greater focus on the possible stress-reduction benefits of horticultural therapy and activities.

These studies have reached inconsistent conclusions, with some studies showing significant effects of horticultural therapy on reducing people's stress levels (Pálsdóttir et al., 2013; Han et al., 2018; Lee et al., 2018b) and others showing non-significant effects (Tu et al., 2020; Wei et al., 2020; Chalmin-Pui et al., 2021). A meta-analysis can synthesize new findings convincingly from previous studies on the same topic (Glass, 1977), while many of the current literature reviews are topic-specific [cognitive function (Tu and Chiu, 2020), depressive symptoms (Zhang et al., 2022), and psychosocial wellbeing (Spano et al., 2020)] or population-specific [the elderly (Wang et al., 2022), people with dementia (Zhao et al., 2020), and people with schizophrenia (Lu et al., 2021)]. Besides, given that differences in the study population, interventions of horticultural therapy, and environmental settings could affect the effectiveness, subgroup analysis is needed for the effect of stress reduction in these areas, which as far as we know has not been addressed in current literature reviews. Therefore, our study included studies with all stress-related physiological and psychological indicators and assessed the stress-reduction effects using a meta-analysis as well as a further subgroup analysis to provide a comprehensive picture of the stress-reduction effects of horticultural therapy.

The aims of this study are to (1) identify the physiological and psychological impacts of horticultural therapy on stress reduction; (2) compare the impact of different groups of people; (3) evaluate the impact of various environmental settings; (4) evaluate the impact of various types of intervention. At the same time, we contrived to develop a theoretical framework that could further serve as a reference for future research as well as our efforts in stress-reduction-related horticultural therapy programs.

## 2. Methods

This quantitative systematic review with meta-analysis was conducted based on Preferred Reporting Items for Systematic Reviews and Meta-Analyses (PRISMA) guidelines (Moher et al., 2009). PRISMA checklist is presented in Appendix A.

### 2.1. Search strategy

We searched relevant studies in six electronic bibliographic databases including PubMed, Cochrane Library, Embase, Web of Science, China National Knowledge Infrastructure, and VIP Data. The search was undertaken by combining search terms for horticultural therapy and stress, with multiple synonymous terms, such as “gardening” and “pressure”. All databases were searched from inception to January 2023. Detailed search steps are presented in Appendix B.

### 2.2. Inclusion criteria and exclusion criteria

Table 1 outlines the inclusion/exclusion criteria, according to the population, intervention, comparison, outcomes, and study design (PICOS).

**Table 1:** Description of the inclusion/exclusion criteria.

**Search strategy**	**Details**
Inclusion criteria	P: No restrictions on the population
	I: Horticultural therapy/gardening
	C: No restrictions on control group
	O: Stress-related physiological and psychological indicators
	S: Randomized controlled trials (RCTs) and quasi-experimental studies
Exclusion criteria	S: Non-original papers (opinion papers, review articles, commentaries, letters, protocols, and reports without quantitative data)
Language filter	English or Chinese
Time filter	Until January 2023
Database	PubMed, Cochrane Library, Embase, Web of Science, China National Knowledge Infrastructure, and VIP Data

Studies normally utilized physiological and psychological indicators to assess the outcomes of stress-reduction effects. Physiological indicators typically include blood pressure (systolic blood pressure and diastolic blood pressure), pulse pressure, saliva cortisol levels, salivary α-amylase (sAA), pulse rate (BPM), heart rate variability (HRV), electroencephalography (EEG), skin conductance (SC), skin temperature (SKT), facial thermal imaging, etc. Psychological indicators were mainly assessed by standardized tests including the Perceived Stress Scale (PSS), the Stress and Crisis Inventory (SCI-93), the Stress Response Scale (SRS-18), the Depression Anxiety Stress Scale (DASS21), the Labor Occupational Pressure Scale, the Geriatric Depression Scale (GDS-30), the Psychosocial wellbeing Index Short Form (PWI-SF), 4T-PROs-Stress, Rehabilitation Stress Scales, etc.

### 2.3. Study selection, data extraction and analysis

We imported all studies into EndNote X8. Two independent reviewers assessed the studies based on the inclusion and exclusion criteria after removing duplicate studies. A third reviewer would be brought in when two independent reviewers had divergent opinions.

We first read the title and abstract of each study, followed by a full-text screening work to decide if it should be included in the analysis. We extracted the following information from each study: (1) basic information, including the research title, first author, and publication year; (2) basic characteristics of the research subjects, including the sample size, age, and gender distribution of people included in each group; (3) details of intervention of horticultural therapy, including intervention activities, duration and settings; (4) critical elements of bias risk assessment; and (5) the outcome indicators.

We pooled the information of the individual studies in Revman5.4 software and R 4.0.3 (R Core Team, 2020) using the “meta” package. Researchers employed a random-effects model to account for study heterogeneity and effect sizes. We employed standardized mean differences (SMDs) because of the various indicators of the stress-relieving outcomes adopted in different studies. The data was compiled using 95 % confidence intervals (CIs). We employed standard I^2^ tests to measure statistical heterogeneity, and we ran a sensitivity analysis to assess the reproducibility and stability of the results. Forest plots were used to visualize the results. Funnel plots were created to visually evaluate publication bias, while Egger's regression test was used to statistically evaluate publication bias.

We also used subgroup analysis to investigate the effects of differences in participants, environmental settings, and interventions of horticultural therapy, accounting for a total of 11 subgroups. As for the participant-related subgroups, we coded their stressors (from education vs. occupation vs. rehabilitation), age, gender, and nationality. The subgroup of environmental settings was coded as indoor, outdoor, combined, and virtual settings. We then categorized the outdoor settings into therapeutically and non-therapeutically designed environments based on the aims and intentions of the design, and we also divided the outdoor settings into farms, gardens, campus, and parks in which horticultural therapy was carried out, to further investigate which kind of outdoor environment could be more effective in stress reduction. We coded the intervention-related subgroups according to the types of activities, duration, frequency, and course. This facilitates researchers and practitioners in developing more effective activities for horticultural therapy.

### 2.4. Risk of bias assessment

Two independent reviewers critically assessed the quality of the eligible studies. To assess the risk of bias in the included studies with RCT designs, we utilized the RCT-specific bias risk assessment tool in the Cochrane handbook for systematic reviews of treatment (Higgins et al., 2011), which assesses randomization procedure biases, allocation concealment, and selective reporting. We used the Joanna Briggs Institute (JBI) critical appraisal tools to assess studies with quasi-experimental designs.

## 3. Results

### 3.1. Study selection

**Figure 1:**
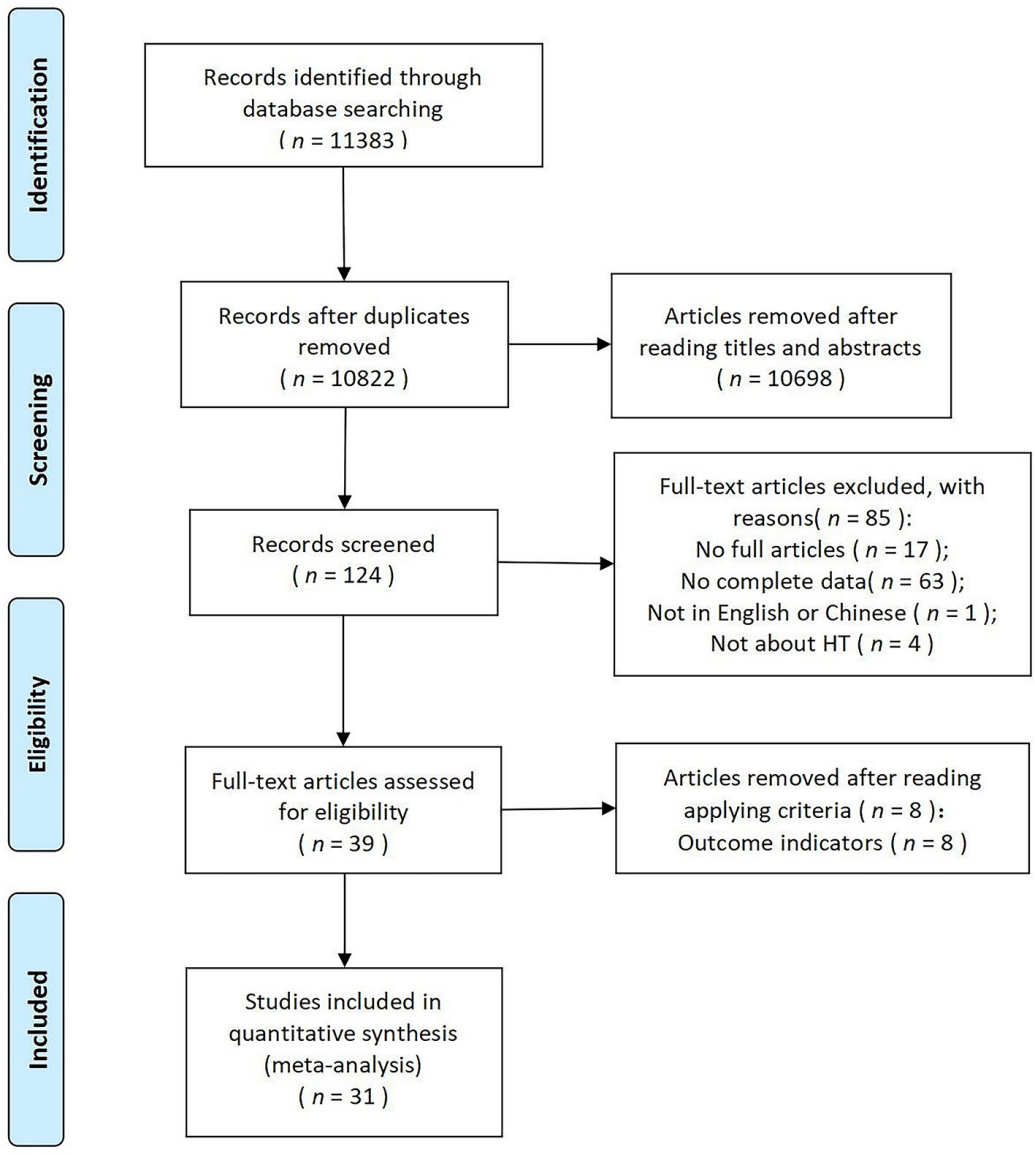
Flow diagram for the systematic review process.

Figure 1 outlines the evaluation procedure. We originally yielded a total of 11,383 articles from PubMed (*n* = 269), Embase (*n* = 1,342), Cochrane Library (*n* = 8), Web of Science (*n* = 9,150), China National Knowledge Infrastructure (*n* = 441), and VIP Data (*n* = 173). Five hundred sixty-one articles were eliminated due to duplication, and 10,698 were removed after screening the titles and abstracts. Of the remaining 124 studies, 17 were removed because the full text was not available, 63 because they lacked comprehensive data, four because they were off-topic, and one because it was not in English or Chinese. Eight studies were further removed because the outcome indicators were irrelevant to stress reduction and detailed reasons are presented in Appendix C. There were 31 studies included in our final analysis (Kam and Siu, 2010; Gonzalez et al., 2011; Hawkins et al., 2011; Van Den Berg and Custers, 2011; Pálsdóttir et al., 2013; Chen et al., 2015; Lee et al., 2015, 2018a,b, 2022; Dewi et al., 2017; Huang et al., 2017; Park et al., 2017a,b; Han et al., 2018; Hassan et al., 2019; Shao et al., 2020; Siu et al., 2020; Tao et al., 2020, 2022; Tu et al., 2020; Wei et al., 2020; Chalmin-Pui et al., 2021; Gong and Chen, 2021; Kim et al., 2021; Meore et al., 2021; Szczepańska-Gieracha et al., 2021; Chan et al., 2022; Curzio et al., 2022; Du et al., 2022; Odeh et al., 2022).

### 3.2. Characteristics of the studies

Appendix D summarizes the characteristics of the studies included in our analysis, of which 21 were quasi-experimental studies and 10 were randomized controlled trials. The reported studies were published between 2010 and 2022, with slightly more articles published in 2020 (*n* = 5), 2021 (*n* = 5) and 2022 (*n* = 6). The sample size ranged from 8 to 113 (1,036 in total). Experimental and control group activities, detailed settings and performers are presented in Appendix E.

#### 3.2.1. Participants

The participants' ages ranged from 7 to 93 years. In the case of gender, most studies involved both male and female participants, with two studies only involving males and seven only females. Furthermore, the various studies were conducted in 10 countries, with the majority in Asia (22 studies, 13 in China, seven in Korea, and two in Japan), followed by Europe (seven studies, two in the UK and one each in Italy, Sweden, the Netherlands, Poland, and Norway), with two study from North America (the USA). Most reported studies did not identify the stressors, except that one study identified participants' stressors from rehabilitation, two studies from education, and two studies from occupation.

#### 3.2.2. Settings

Fourteen studies conducted the intervention of horticultural therapy in indoor settings, 11 in outdoor settings (three in farms, six in gardens, one in campus and one in parks), four in a combination of indoor and outdoor settings, and one involved virtual reality. One study did not mention the settings.

#### 3.2.3. Interventions

The interventions of horticultural therapy, mainly refer to horticultural activities in this analysis, including transferring plants, tasting and smelling, handcrafting activities, flower arrangement, transplanting plants, potting activities, soil-mixing activities, harvesting activities, planting and sowing activities, walking and meditation. The intervention also differed in terms of duration (three minntes to 210 min), total duration (3–10,080 min), and frequency (two to three times a month to four times a week).

### 3.3. Risk of bias

**Figure 2:**
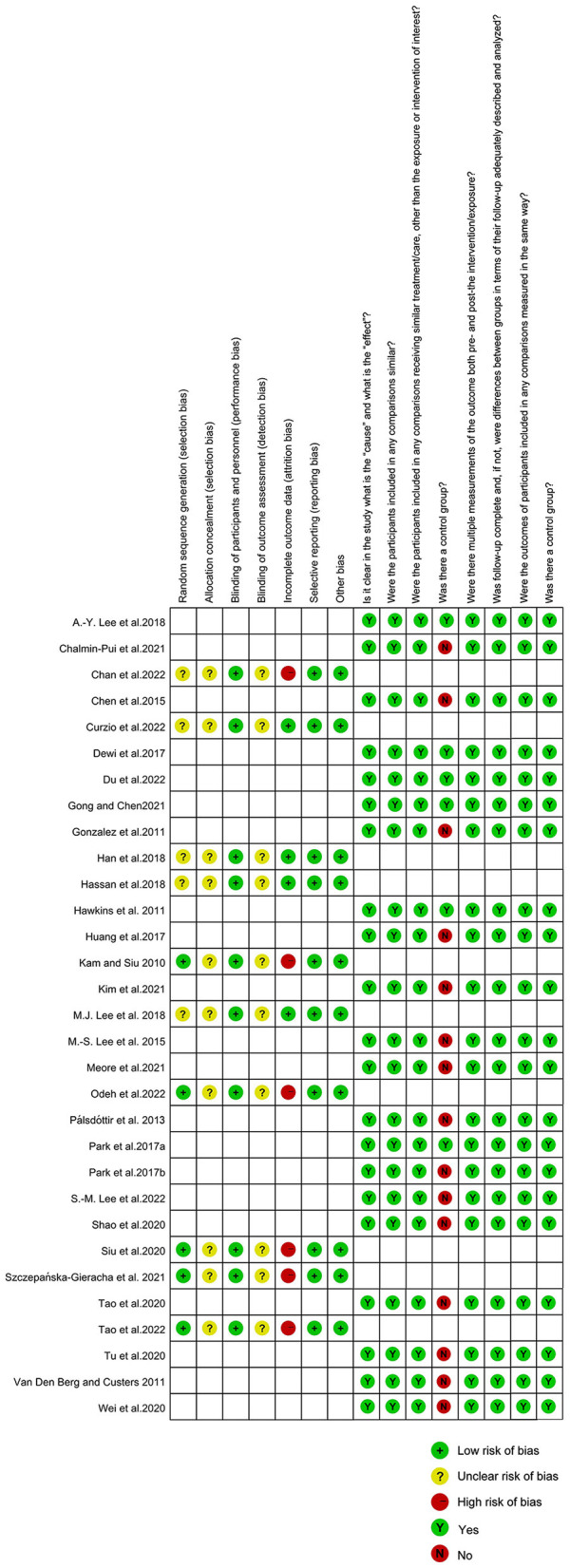
Summary of the risk of bias of included studies.

Allocation concealment and outcome assessment blinding were rated as unclear risks, whereas five studies did not describe in detail the method of random sequence generation and six studies had instances of participation withdrawal due to incomplete outcome data. The majority of studies were found to be of low risk of bias. We followed the JBI critical appraisal checklist to assess the quasi-experimental studies involved. Figure 2 shows the results of the risk evaluation.

### 3.4. Meta-analysis outcomes

Thirteen quasi-experimental studies and five studies with RCT designs were adopting physiological indicators to assess the stress-reduction effects, while ten quasi-experimental studies and six studies with RCT designs adopted psychological indicators. Therefore, we used SMDs to manage the differences in measurements, and the meta-analysis was estimated under a random-effects model.

**Figure 3:**
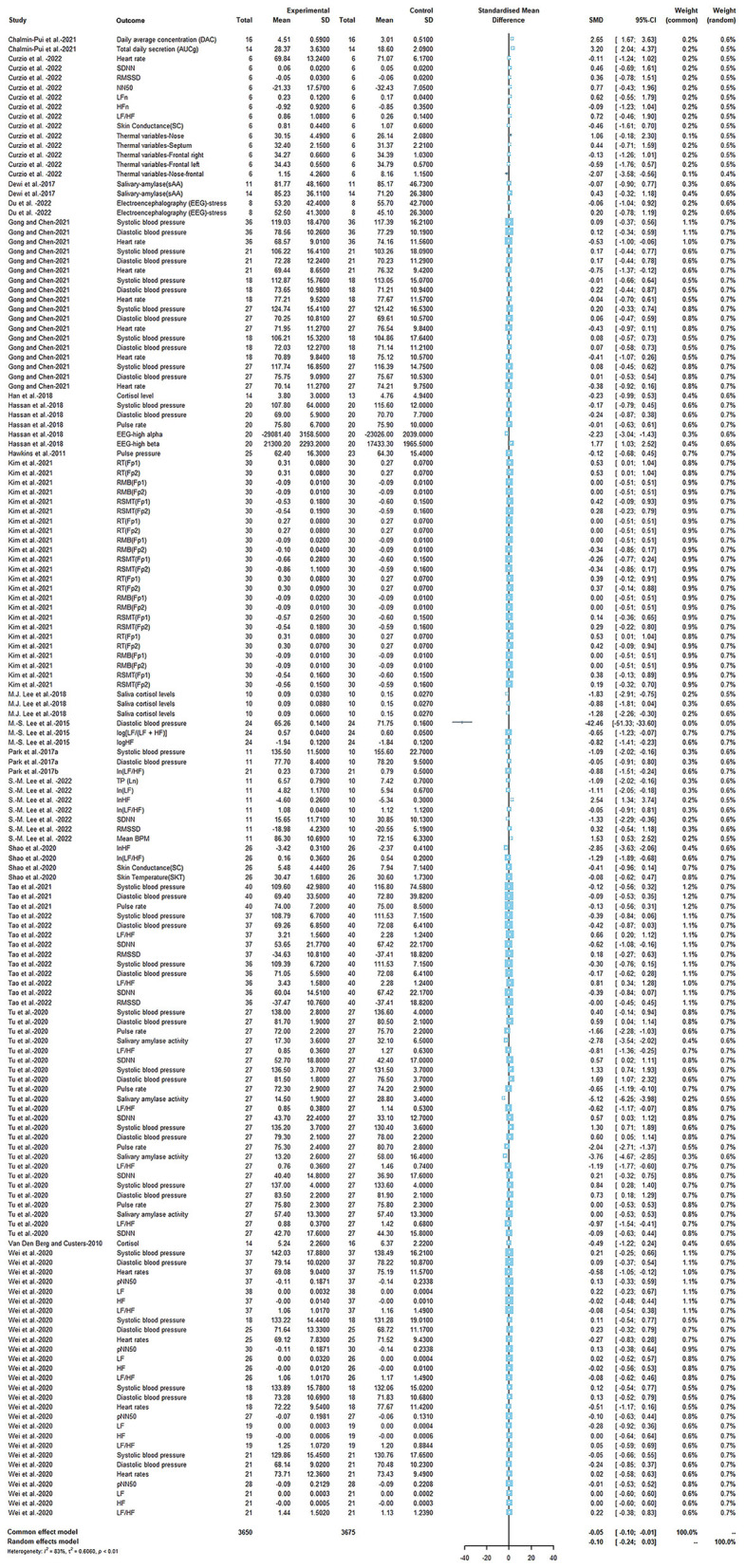
Effects on the physiology indicators.

Figure 3 shows the effects on the physiology indicators, with the outcomes slightly varied (SMD = −0.10, 95% CI [−0.24, 0.03], *p* = 0.13, *I*^2^ = 83%) in terms of the influence of horticultural therapy on stress. We detected significant differences in the sensitivity analyses when removing (Tu et al., 2020) (SMD = −0.05, 95% CI [−0.15, 0.05], *p* = 0.33, *I*^2^ = 73%).

**Figure 4:**
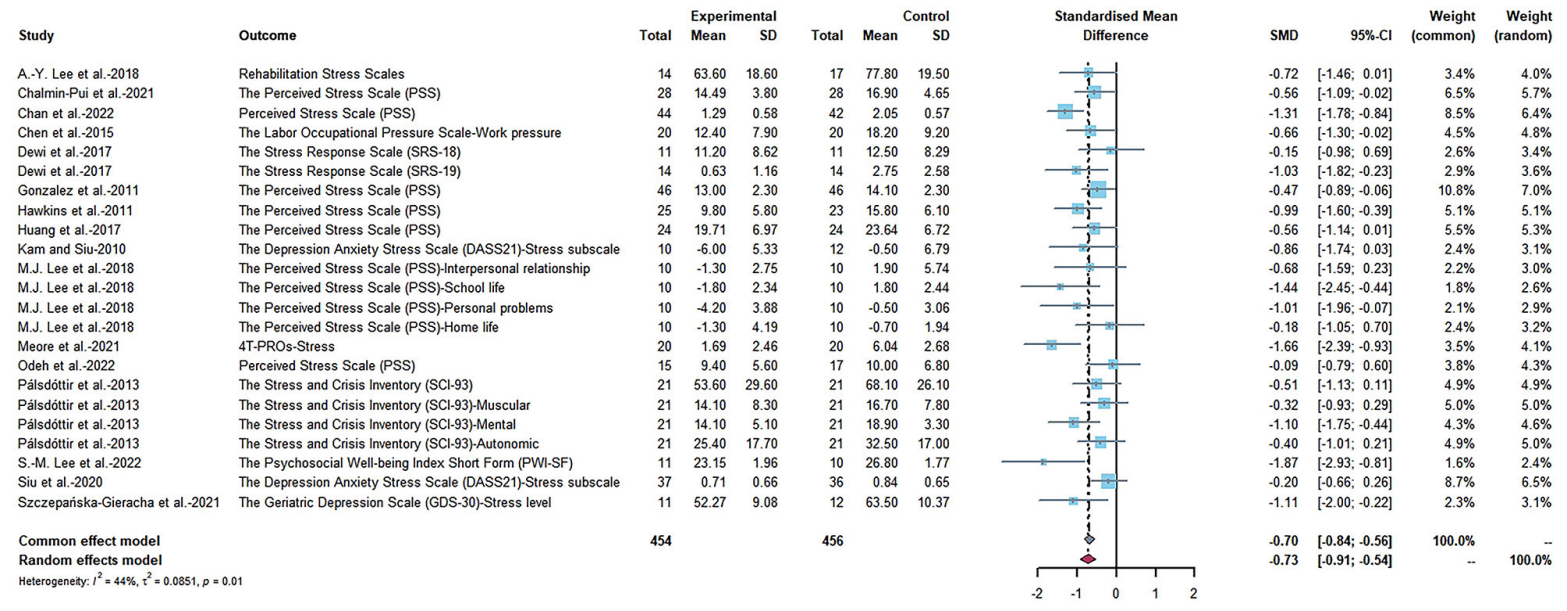
Effects on the physiology indicators.

In comparison, the psychological effectiveness was more significant (SMD = −0.73, 95% CI [−0.91, −0.54], *p* < 0.0001, *I*^2^ = 44%), as shown in Figure 4. We removed all the studies included in this meta-analysis one by one. When the study of Meore et al. (2021) and Chan et al. (2022) was removed, the results showed that heterogeneity was reduced (SMD = −0.68, 95% CI [−0.86, −0.51], *p* < 0.0001, *I*^2^ = 35%; SMD = −0.68, 95% CI [−0.86, −0.50], *p* < 0.0001, *I*^2^ = 35%).

### 3.5. Subgroup analysis outcomes

**Figure 5:**
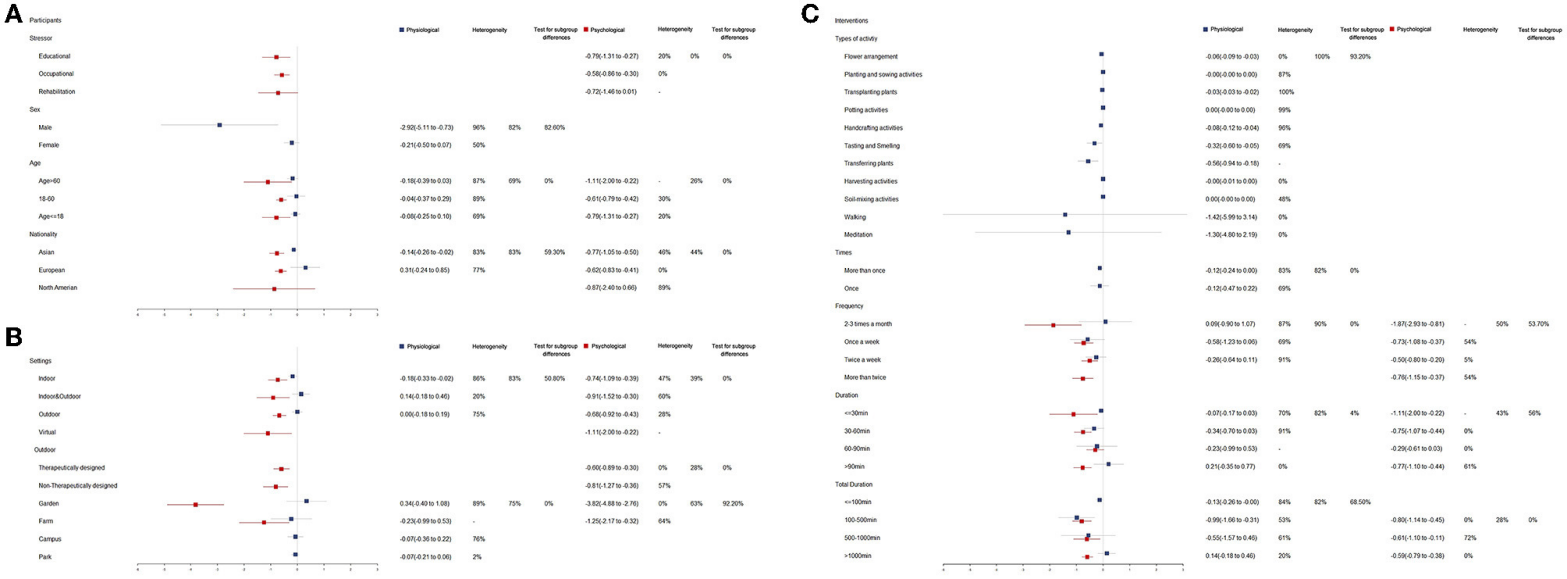
**(A)** Results of participant-related subgroup analysis. **(B)** Results from setting-related subgroup analysis. **(C)** Results from intervention-related subgroup analysis.

We used subgroup analysis to investigate the effects of differences in participants, environmental settings, and interventions of horticultural therapy. Figure 5 shows the subgroup analysis outcomes.

#### 3.5.1. Participants

##### 3.5.1.1. Stressor

Horticultural therapy efficiently lowered stress related to educational stressors (SMD = −0.79), compared to occupational and rehabilitation stressors (SMD = −0.58 and SMD = −0.72, respectively) in psychological indicators.

##### 3.5.1.2. Sex

Males (SMD = −2.92) obtained better stress-relieving effects than females (SMD = −0.21) in physiological indicators.

##### 3.5.1.3. Age

Horticultural therapy was most effective in reducing stress in people aged over 60 (SMD = −0.18 in physiological indicators; SMD = −1.11 in psychological indicators), followed by people aged under 18 (SMD = −0.08 in physiological indicators; SMD = −0.79 in psychological indicators).

##### 3.5.1.4. Nationality

Participants from Asia had a better stress reduction experience in horticultural therapy (SMD = −0.14) in terms of physiological indicators, while participants from North America had a better stress reduction experience in terms of psychological indicators (SMD = −0.87).

#### 3.5.2. Settings

The results confirmed that the indoor setting had the best decompression effect (SMD = −0.18) in terms of physiological indicators, while the virtual environment constituted the most effective in terms of psychological indicators (SMD = −1.11).

The results show that the non-therapeutically designed settings had a better decompression effect (SMD = −0.81) than therapeutically designed settings (SMD = −0.60). The garden settings were more effective in terms of psychological indicators (SMD = −3.82), while the farm settings were more effective in terms of physiological indicators (SMD = −0.23).

#### 3.5.3. Interventions

##### 3.5.3.1. Type of activities

We included 10 studies in the activity-specific subgroup analysis, among which five reported studies involved multiple horticultural activities as interventions (Lee et al., 2018b; Tu et al., 2020; Wei et al., 2020; Gong and Chen, 2021; Kim et al., 2021), and seven studies involved single horticultural activity as interventions (Van Den Berg and Custers, 2011; Lee et al., 2015; Park et al., 2017b; Hassan et al., 2019; Shao et al., 2020; Tao et al., 2020; Du et al., 2022). The results revealed that walking (SMD = −1.42), meditation (SMD = −1.30), transferring plants (SMD = −0.56), and tasting and smelling (SMD = −0.32) were more effective in reducing stress, while other types of activity had limited or no stress-relieving effect.

##### 3.5.3.2. Times

The results show that the decompression effect was independent of the times of the intervention (SMD = −0.12).

##### 3.5.3.3. Frequency

The once-a-week session was the most effective in terms of physiological indicators (SMD = −0.58), while the 2-to-3-times-a-month session was the most effective in psychological indicators (SMD = −1.87).

##### 3.5.3.4. Duration

Physiological indicators showed a duration of 30–60 min is the most effective (SMD = −0.34); in comparison, psychological indicators showed a duration of fewer than 30 min is the most effective (SMD = −1.11).

##### 3.5.3.5. Total duration

The total duration of 100–500 min is the most effective in both physiological (SMD = −0.99) and psychological indicators (SMD = −0.80).

### 3.6. Results of publication bias

**Figure 6:**
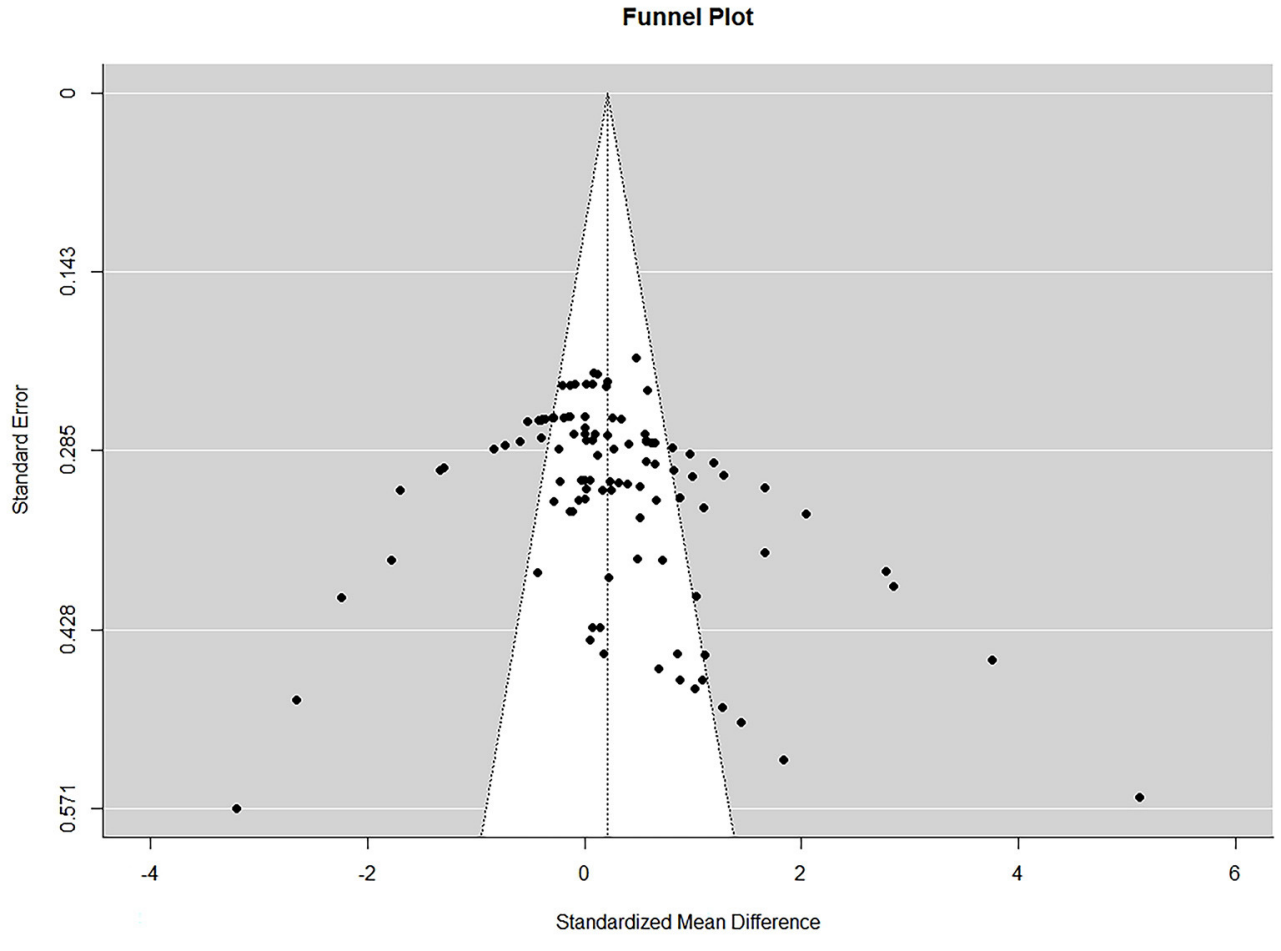
Funnel plot.

Funnel plots were created to visually evaluate publication bias. The funnel plot showed an approximate symmetrical distribution of study effect size, which suggests that there might not be any publication bias (Figure 6). Furthermore, Egger's regression test was used to statistically evaluate publication bias. The bias coefficient of Egger's test was < 0.0001, so there was a possibility of publication bias.

## 4. Discussion

### 4.1. Participants' stressors and characteristics

Stress is often linked to complicated stressors (Chauhan et al., 2015), such as individual factors, relationship characteristics, health, work and education, community, finances, and the environment (Brannen et al., 2009). There was a limited number of studies identifying participants' stressors. Future research with clearly defined stressors is needed to develop stress reduction strategies for specific stressors and to improve the practice of horticulture therapy. Gender, ethnicity, and age have an impact on people's stressors and stress levels.

People with educational stressors obtained better stress reduction benefits in horticultural therapy activities. These activities transferred students' focus from daily stressful situations to plants, allowing them to experience happy feelings (Oh et al., 2020).

Males obtained better stress-relieving effects than females. Our results were consistent with other empirical studies that the self-esteem levels and emotional state of males increased more significantly than females after green exercise (Barton and Pretty, 2010). Females consider stressors as more threatening (Ptacek et al., 1992) and adopt more emotion-focused responses compared to males (Matud, 2004), making it more difficult to benefit from the stress-relieving effects of horticultural therapy.

People of different ethnic groups also differed in their level of stress (Wei et al., 2011; Hamamura and Laird, 2014) as well as their stress management strategies (Lam and Zane, 2004; Sawaumi et al., 2015). This could explain the fact that better stress reduction on physiological indicators was achieved by Asian participants, while better stress reduction on psychological indicators was achieved by North American participants.

People over 60 years old obtained better stress-reduction benefits from horticultural therapy. Long-term stressors can be harmful to people's health, especially elderly people (Schneiderman et al., 2005; Hurst et al., 2013). A review found that horticulture therapy could improve the physical and psychological health of older persons, which is consistent with our findings (Lin et al., 2022). Gardening appears to activate many important protective mechanisms for active and healthy aging. Therefore, the elderly, particularly in nursing homes and retirement communities, could be provided with more opportunities for horticulture therapy.

### 4.2. Characteristics and selection of intervention settings

The settings for horticultural therapy were essential, and they also had an important influence on the therapeutic benefits (Huxmann, 2016). Our results suggested that indoor and virtual environments were more effective in stress reduction than outdoor settings, which might be somewhat inconsistent with previous studies. This is possible because indoor and virtual environments had a relatively homogeneous and quiet atmosphere which were not likely to be affected by other distracting factors (e.g., other people, other animals, weather, temperature, sun exposure, noise, etc.,) (Guo et al., 2020). In other studies, for example, Brooks and colleagues argue that actual and virtual nature interactions were both beneficial to moods, though actual nature interactions yielded better outcomes (Brooks et al., 2017). Therefore, we encourage people to connect with “First Nature” and “Second Nature” as much as possible. From a practical standpoint, we recommend environments with both indoor and outdoor attributes, especially considering people with limited mobility and weather conditions that prevent outdoor activities.

We found that conducting horticultural therapy activities in gardens had greater effects on psychological indicators. Many studies found that gardens were more suitable environments for stress reduction (Kohlleppel et al., 2002; Coventry and White, 2018; Ulrich et al., 2020) than parks and green views in terms of psychological health (Marques et al., 2021). Meanwhile, the high biodiversity of gardens had a huge benefit in increasing the stress-relieving impact (Keniger et al., 2013; Oh et al., 2020).

### 4.3. Characteristics and effectiveness of the interventions

The lack of direct comparisons between the various activities made it hard to verify whether one activity contributed to the reported effect (Murroni et al., 2021). This question has been answered in our subgroup analysis. Activities that activate the five senses, such as walking, meditation, transferring plants, and tasting and smelling were more effective. At the same time, it is important to consider the different intensities of activities for different groups of people when choosing the types of activity (Park et al., 2014; Lee et al., 2021), with a focus on low and medium-intensity activities.

It is also a key issue to determine the duration and frequency of horticultural therapy programs (Tu and Chiu, 2020). The 30–60 min session was more effective in physiology indicators and the < 30 min session was more effective in psychological indicators, which could achieve the stress-reduction goals and at the same time not make participants feel bored during the session. A total duration of 100–500 min could be more beneficial by maintaining the appeal and uniqueness while attracting people's attention and willingness to engage in the cyclical process of treatment.

### 4.4. A theoretical framework

Our findings supported the positive effect of horticultural therapy on stress reduction. Educational stressors achieved better results with horticultural therapy interventions. Seniors over 60 and males had a better stress reduction experience in horticultural therapy. Indoor and virtual areas were the most effective setting for horticultural therapy and we believed that a combination of outdoor and indoor areas was the optimal setting for horticultural therapy. At the same time, a total duration of 100–500 min provided better effects of stress reduction.

We developed a theoretical framework for horticulture therapy in terms of its stress-reduction effects on physiological and psychological indicators based on “Participants-Settings-Interventions” to provide a reference for future horticultural therapy activities (Table 2).

**Table 2:** Participants-settings-interventions stress reduction theoretical framework.

	**Physiology**	**Psychological**
**Participants**		
Stressor	–	Educational
Sex	Male	–
Age	Age > 60	Age > 60
Nationality	Asian	North American
**Settings**		
	Indoor	Virtual
	–	Non-Therapeutically designed (Outdoor)
	Farm (Outdoor)	Garden (Outdoor)
**Interventions**		
Types of activity	Walking	–
Frequency	Once a week	2–3 times a month
Duration	30–60 min	≤ 30 min
Total duration	100–500 min	100–500 min

We also identified several limitations in this literature review. First, studies that were not published in English or Chinese were not included in this review and generalizability may be limited. Second, the lack of randomized controlled trials of high quality, though difficult to perform, also limited our outcomes. Only ten out of 31 reported studies were randomized controlled trials, let alone the participant withdrawal in several RCT studies. Finally, the number of articles in the study, the sample size of these articles, and the heterogeneity between studies would have affected the results of the subgroup analysis. Moreover, due to the lack of specific data in some of the included studies, we were unable to conduct a subgroup analysis of these studies.

## 5. Conclusion

Our meta-analysis found evidence of the beneficial effects of horticultural therapy on stress reduction. We developed a comprehensive theoretical framework that explains the design strategies for horticulture therapy activities in terms of the environmental settings and the interventions (types of activity, duration, frequency, and course) for diverse populations with varied stressors.

We have to pay more attention to the ongoing effect, especially when the program lasts for a longer period of time. Besides, future randomized controlled trials should clearly describe the blind evaluation, suitable follow-up duration, study size calculation, and basic descriptive statistics (e.g., means and standard deviations), all of which are essential for readers and follow-up research.

A comprehensive guide to the operation of horticultural therapy is needed in order to provide realistic therapeutic interventions with sufficient scientific value and clinical relevance. Our results contribute to addressing the question of how horticultural therapy activities can be organized to maximize the stress-relieving effects on different groups of people, to improve their physical and mental health as well as their quality of life.

## Data availability statement

The original contributions presented in the study are included in the article/Supplementary material, further inquiries can be directed to the corresponding author.

## Author contributions

SL: data curation and writing—original draft preparation. SL, FX, JL, and MX: writing—review and editing. FX: supervision. All authors have read and agreed to the published version of the manuscript.
