# Supplementary material for: Horticultural therapy for stress reduction: A systematic review and meta-analysis
Source: Front Psychol. 2023 Jul 26;14:1086121. doi: 10.3389/fpsyg.2023.1086121 (PMC10411738; doi:10.3389/fpsyg.2023.1086121)
Supplement: Supplementary file 2 [file Table_2.docx]

**Table 2**

Pubmed was searched on 23/1/29

| **No.** | **Search terms** | **Hits** |
| --- | --- | --- |
|  | "Gardening"[Mesh] | 1167 |
|  | "Horticultural Therapy"[Mesh] | 93 |
|  | horticultur*[Title/Abstract] | 4598 |
|  | plant[Title/Abstract] | 462822 |
|  | garden[Title/Abstract] | 9971 |
|  | farm[Title/Abstract] | 42476 |
|  | ("Gardening"[Mesh]) OR("Horticultural Therapy"[Mesh]) OR(horticultur*[Title/Abstract])OR(garden[Title/Abstract])OR (farm[Title/Abstract]) | 57333 |
|  | "Stress, Psychological"[Mesh] | 150649 |
|  | "Pressure"[Mesh] | 119060 |
|  | (("Stress, Psychological"[Mesh]) OR("Pressure"[Mesh])) AND(("Gardening"[Mesh]) OR("Horticultural Therapy"[Mesh]) OR (horticultur*[Title/Abstract])OR(garden[Title/Abstract]) OR (farm[Title/Abstract])) | 269 |

Embase was searched on 23/1/29

| **No.** | **Search terms** | **Hits** |
| --- | --- | --- |
|  | 'gardening'/exp | 2182 |
|  | 'horticultural therapy'/exp | 179 |
|  | horticultur*:ab,ti | 4314 |
|  | garden:ab,ti | 10603 |
|  | farm:ab,ti | 46506 |
|  | 'physiological stress'/exp | 475131 |
|  | 'pressure'/exp | 116471 |
|  | #1 OR #2 OR #3 OR #4 OR #5 | 62658 |
|  | #6 OR #7 | 590070 |
|  | #8 AND #9 | 1342 |

Cochrane Library was searched on 23/1/29

| **No.** | **Search terms** | **Hits** |
| --- | --- | --- |
|  | MeSH descriptor: [Gardening] in all MeSH products | 42 |
|  | MeSH descriptor: [Horticultural Therapy] explode all trees | 20 |
|  | (horticultur*):ti.ab,kw | 144 |
|  | (garden):ti,ab,kw | 340 |
|  | (farm):ti,ab,kw | 443 |
|  | #1 OR #2 OR #3 OR #4 OR #5 | 932 |
|  | MeSH descriptor: [Pressure] explode all trees | 3713 |
|  | MeSH descriptor: [Stress, Psychological] explode all trees | 6960 |
|  | #7 OR #8 | 10662 |
|  | #6 AND #9 | 8 |

Web of Science was searched on 23/1/29

| **No.** | **Search terms** | **Hits** |
| --- | --- | --- |
|  | (TI=(stress) OR AB=(stress) OR TI=(pressure) OR AB=(pressure)) AND (OA==("OPEN ACCESS")) AND (TI=(horticultural therapy) OR AB=(horticultural therapy) OR TI=(garden) OR AB=(garden) OR TI=(farm) OR AB=(farm) OR TI=(horticultur*) OR AB=(horticultur*) OR TI=(gardening) OR AB=(gardening)) AND (OA==("OPEN ACCESS")) | 9150 |

CNKI was searched on 23/1/29

| **No.** | **Search terms** | **Hits** |
| --- | --- | --- |
|  | ((((主题%=‘园艺’ or 题名%=‘园艺’ or v_subject=xls(‘园艺’)) OR (主题%=‘农’ or 题名%=‘农’ or v_subject=xls(‘农’))) OR (主题%=‘花园’ or 题名%=‘花园’ or v_subject=xls(‘花园’))) AND (主题%=‘压力’ or 题名%=‘压力’ or v_subject=xls(‘压力’))) | 441 |

VIP Data was searched on 23/1/29

| **No.** | **Search terms** | **Hits** |
| --- | --- | --- |
|  | ((M=园艺 OR M=农) OR M=花园) AND M=压力 | 173 |
