# Supplementary material for: Horticultural therapy for stress reduction: A systematic review and meta-analysis
Source: Front Psychol. 2023 Jul 26;14:1086121. doi: 10.3389/fpsyg.2023.1086121 (PMC10411738; doi:10.3389/fpsyg.2023.1086121)
Supplement: Supplementary file 3 [file Table_3.docx]

**Table 3**

Excluded studies with reasons

| **No** | **Studies** | **Outcomes** |
| --- | --- | --- |
| 1 | Nature-Based Stress Management Course for Individuals at Risk of Adverse Health Effects from Work-Related Stress—Effects on Stress Related Symptoms, Workability and Sick Leave | The Shirom-Melamed Burnout Questionnaire (SMBQ), the Work Ability Index (WAI) and sleep quality |
| 2 | Using Nature-Based Rehabilitation to Restart a Stalled Process of Rehabilitation in Individuals with Stress-Related Mental Illness | The Shirom-Melamed Burnout Questionnaire (SMBQ), the Beck Depression Inventory (BDI-II), the Beck Anxiety Inventory and The Psychological General Well-Being Index (PGWB) |
| 3. | ‘Everything just seems much more right in nature’: How veterans with post-traumatic stress disorder experience nature-based activities in a forest therapy garden | The veterans’ experiences of the NBAs and nature |
| 4 | Predictors of non-pharmacological intervention effect on cognitive function and behavioral and psychological symptoms of older people with dementia | The Barthel Index and Lawton–Brody Instrumental Activities of Daily Living (IADL) Scale, the Mini-Mental Status Examination (MMSE) and the Neuropsychiatric Inventory (NPI) |
| 5 | The effect of horticultural therapy on the quality of life of palliative care patients | The QOLC-E Questionnaire |
| 6 | A Diagnostic Post-Occupancy Evaluation of the Nacadia® Therapy Garden | The EQ-VAS (self-estimated general health) |
| 7 | Impact of Nurses Taking Daily Work Breaks in a Hospital Garden on Burnout | The Maslach Burnout Inventory, a Present Functioning Visual Analogue Scale |
| 8 | Urban farming: a non-traditional intervention for HIV-related distress | The PHQ-9, y the Generalized Anxiety Disorder-7 and the SF-12 |
