# Supplementary material for: Horticultural therapy for stress reduction: A systematic review and meta-analysis
Source: Front Psychol. 2023 Jul 26;14:1086121. doi: 10.3389/fpsyg.2023.1086121 (PMC10411738; doi:10.3389/fpsyg.2023.1086121)
Supplement: Supplementary file 4 [file Table_4.docx]

**Table 4**

| **Author &Publication Year** | **Country** | **Stressor** | **Population** | | | | **Settings** | **Intervention** | | | | | **Measurement and Outcomes** |
| --- | --- | --- | --- | --- | --- | --- | --- | --- | --- | --- | --- | --- | --- |
|  |  |  | **Subjects** | **Participants**  **E/C**  **(drop out)** | **Age E/C** | **Male/Female** |  | **Duration**  **(min)** | **Frequency** | **Follow-up** | **Times** | **Total Duration**  **(min)** |  |
| A.-Y. Lee et al., 2018 | Korea | Rehabilitation | stroke patients | 14/17 | 53.4±12.6/56.1±10 | 6/8; 10/7 | Indoor&Outdoor | 60min | Three times a week | Six weeks | 18 | 1080 | Rehabilitation stress survey |
| Chalmin-Pui et al., 2021 | UK | - | Residents | 28 | 18-85 | 15/27 | Outdoor | - | - | 12 weeks | - | - | The Perceived Stress Scale (PSS); Saliva cortisol levels |
| Chen et al., 2015 | China | Occupational | Nursing Staff | 20 | 32.3±5.3 | 0/20 | Indoor | 60min | Once a week | Four weeks | 4 | 240 | The Labor Occupational Pressure Scale |
| Dewi et al., 2017 | Japan | - | Adults with and without Mental Disabilities | 11/14 | 37±10/32±19 | 21/7 | Indoor&Outdoor | 120min | Once a week | 27 weeks | 27 | 3240 | Salivary α-amylase(sAA); Psychological stress response scale (SRS-18) |
| Gonzalez et al., 2011 | Norway | - | Depression | 46 | 46.3±11.6 | 10/36 | Outdoor | 180min | Twice a week | 12 weeks | 24 | 4320 | The Perceived Stress Scale (PSS) |
| Han et al., 2018 | Korea | - | Elderly people with mental health problems | 14/14 | 80.1±2.9/77.4±5.98.49 | 1/13; 3/11 | Outdoor | 90min | Once a week | Ten weeks | 10 | 900 | Cortisol levels |
| Hassan et al., 2019 | China | Educational | Chinese adult males and females from Sichuan Agricultural University | 20/20 | 19.3±1.3 | Not mentioned | Indoor | 10min | - | - | 1 | 10 | Blood pressure; pulse rate; electroencephalography (EEG) |
| Hawkins et al., 2011 | UK | - | Allotment gardeners | 23/25 | 72.9±6.9/65.7±9.1 | 3/20; 17/8 | Outdoor | - | - | - | - | - | The Perceived Stress Scale (PSS); Pulse pressure |
| Huang et al., 2017 | China | - | Family caregiver | 8(6) | 49.25±8.21 | 1/7 | Indoor | 60-120min | Twice a week | Three weeks | 6 | 540 | The Perceived Stress Scale (PSS) |
| Kam and Siu, 2010 | China | - | Persons with psychiatric illness | 10(2)/12 | 45.3±10.38/43.3±11.7 | 8/4; 9/3 | Outdoor | 60min | - | Ten days | 10 | 600 | The Depression Anxiety Stress Scale (DASS21) |
| Kim et al., 2021 | Korea | - | Elementary school students | 30 | 11.3±1.3 | 11/19 | Indoor | 3min | - | - | 1 | 3 | Electroencephalography (EEG) |
| M. J. Lee et al., 2018 | Korea | Educational | Elementary school children | 10/10 | 10.4/10.5 | 0/10; 0/10 | Indoor | 40min | Once a week | Nine weeks | 9 | 360 | The Perceived Stress Scale (PSS); Saliva cortisol levels |
| 1. S. Lee et al., 2015 | Japan | - | Young adults | 24 | 24.9±2.1 | 24/0 | Indoor | 15min | - | - | 1 | 15 | Blood pressure; Heart Rate Variability (HRV) |
| Meore et al., 2021 | USA | - | Veterans with history of suicidality | 20 | 51.25±17.29 | 10/10 | Indoor&Outdoor | 210min | Four times a week | Four weeks | 16 | 3360 | 4T-PROs-Stress |
| Pálsdóttir et al., 2013 | Swedish | Occupational | psychiatric diagnosis of adjustment disorder and reaction to severe stress (ICD F43); depression (ICD F32.0, ICD F32.1) | 21 | 29-68/47 | 2/19 | Outdoor | 210min | Four times a week | 12 weeks | 48 | 10080 | The Stress and Crisis Inventory (SCI-93) |
| Park et al., 2017a | Korea | - | Women over the age of 70 | 11/10 | 80.3±6/81±4.3 | 0/21 | Outdoor | 50min | Twice a week | 7.5 weeks | 15 | 750 | Blood pressure |
| Park et al., 2017b | Korea | - | Male university students | 12/12 | 24.0±2.7 | 24/0 | Indoor | 3min | - | - | 1 | 3 | Heart Rate Variability (HRV) |
| Shao et al., 2020 | China | - | Elementary school students | 26 | 8.12±1.07 | 15/11 | Indoor | 5min | - | - | 1 | 5 | Heart Rate Variability (HRV); Skin Conductance (SC); Skin Temperature (SKT) |
| Siu et al., 2020 | China | - | People with mental illness | 37(4)/36(5) | 50.8±10.5/49.7±8.7 | 20/21; 17/24 | Not mentioned | 75min | Once a week | Eight weeks | 8 | 600 | The Depression Anxiety Stress Scale (DASS21) |
| Szczepańska-Gieracha et al., 2021 | Poland | - | Depressive Symptoms in Late-Life | 11(2)/12 | 70.18±4.87/71.25±4.41 | 0/23 | Virtual | 20min | Twice a week | Four weeks | 8 | 160 | The Geriatric Depression Scale (GDS-30)-stress level |
| Tao et al., 2020 | China | - | Chinese females | 40 | 22.2±0.9 | 0/40 | Indoor | 12min | - | - | 1 | 12 | Blood pressure; Pulse rate |
| Tu et al., 2020 | China | - | The elderly | 27 | 67.9±4.5 | 5/22 | Indoor | 60min | Twice a week | Two weeks | 4 | 240 | Blood pressure; Pulse rate; Salivary amylase activity |
| Van Den Berg and Custers, 2011 | Netherlands | - | healthy plot holders | 14/16 | 58.29±8.49/57±8.49 | 8/22 | Outdoor | 30min | - | - | 1 | 30 | Cortisol |
| Wei et al., 2020 | China | - | The elderly without family members | 37(7) | 79.70±5.67 | 7/29 | Indoor | 30min | - | - | 4 | 120 | Blood pressure; heart rate; Heart Rate Variability (HRV) |
| Gong and Chen, 2021 | China | - | Residents | 40 | Not mentioned | Not mentioned | Outdoor | 30min | - | - | 6 | 180 | Blood pressure; heart rate |
| Chan et al., 2022 | China | - | Community-dwelling older adults | 44/42(4) | Not mentioned | 12/30; 10/34 | Indoor | 120min | Once a week | 8 weeks | 8 | 960 | The Perceived Stress Scale (PSS) |
| Curzio et al., 2022 | Italy | - | Adolescents with Anorexia Nervosa | 6/6 | 14.86±1.92 | 0/12 | Indoor&Outdoor | 45min | Twice a week | 12 weeks | 24 | 1080 | Skin Conductance(SC); Heart Rate(HR); Heart Rate Variability (HRV); facial thermal imaging |
| Du et al., 2022 | China | - | Normal Elderly (NE) and Cognitively Impaired Elderly (CIE) | 16 | 85±8.7 | Not mentioned | Indoor | 10-15min | - | - | 1 | 10-15 | Electroencephalography (EEG) |
| S.-M. Lee et al., 2022 | Korea | - | Residents | 11(15)/10(18) | 66.6±8.1;56.6±5.8 | 0/11; 2/8 | Outdoor | Not mentioned | 2–3 times a month | 9 months | 19 | - | The Psychosocial Well-being Index Short Form (PWI-SF); heart rate variability (HRV) |
| Odeh et al., 2022 | USA | - | Healthy women | 16(5)/20(1) | 32.1±5.1; 32.8±5.6 | 0/16; 0/20 | Indoor | 70min | Twice a week | Four weeks | 8 | 560 | The Perceived Stress Scale (PSS) |
| Tao et al., 2022 | China | - | Residents | 37(3)/40; 36(4)/40 | 18-26, average age 22.5 | Not mentioned | Outdoor | 20min | - | - | 1 | 20 | Blood pressure; heart rate variability (HRV) |
