# Supplementary material for: Horticultural therapy for stress reduction: A systematic review and meta-analysis
Source: Front Psychol. 2023 Jul 26;14:1086121. doi: 10.3389/fpsyg.2023.1086121 (PMC10411738; doi:10.3389/fpsyg.2023.1086121)
Supplement: Supplementary file 5 [file Table_5.docx]

**Table 5**

Experimental and control group activities

| **Studies** | **Activities-E** | **Activities-C** |
| --- | --- | --- |
| A.-Y. Lee et al. (2018) | Horticultural activities including the motions such as reaching–grasping, squatting, stepping, and stooping | Not mentioned |
| Chalmin-Pui et al. (2021) | A horticultural intervention introduced ornamental plants | - |
| Chen et al. (2015) | Growing vegetables, painted potted plants, table Zen gardens and qiandry flowers | - |
| Dewi et al. (2017) | Community garden activities including crop cultivation, seedling preparation, transplanting, planting, weeding, inter-tillage, top-dressing, pillar standing, pest control, harvesting, and cleaning up | - |
| Gonzalez et al. (2011) | Horticultural therapy sessions | - |
| Han et al. (2018) | Plant cultivating activities such as making plant beds, planting transplants, watering, weeding, and harvesting | Indoor activity programs such as crafts, cooking, or game that were provided at the mental health centers |
| Hassan et al. (2019) | Transplanting the plants | Performing a computer task |
| Hawkins et al. (2011) | Carry out their gardening activity on an individual plot of land within a site consisting of many other gardening plots | Perform physical activity inside leisure centers or similar community venues |
| Huang et al. (2017) | Introduction to horticultural therapy, knowledge of plant species, planting techniques, production of related finished products or artefacts, individual programme teaching | - |
| Kam and Siu (2010) | Horticultural programme (a) to teach basic horticultural knowledge and skills, and to develop interests in working with plants, (b) to share relaxing experience and coping strategies through working with plans, (c) to promote sharing and social support among participation. | Conventional workshop training |
| Kim et al. (2021) | Horticultural activities such as harvesting, planting, sowing seeds, and mixing soil | Ball-playing, math, video, paper-folding, reading |
| M.J. Lee et al. (2018) | Flower arrangement (FA), planting (P), and flower pressing (PF) activities | Individual favorite indoor activities |
| M.-S. Lee et al. (2015) | Transplanting of an indoor plant | Working on a computer task |
| Meore et al. (2021) | Seed sowing, seed paper making, seedling thinning and transplanting, harvesting, staking and trellising of plants as well as general garden and plant maintenance | - |
| Pálsdóttir et al. (2013) | Occupational therapy, physiotherapy, psychotherapy, and horticultural therapy | - |
| Park et al. (2017a) | Planning a garden, making a garden plot, planting, sowing, mulching, fertilizing, watering, weeding, harvesting, garden maintenance, and cleaning the garden plot | Not mentioned |
| Park et al. (2017b) | Performed the task with foliage plants | Performed the same task without foliage plants |
| Shao et al. (2020) | Perform a plant-related task, cultivate in pots filled with soil | Perform a mobile game task |
| Siu et al. (2020) | Focused on basic horticulture knowledge and skills; use plants, fruits, and herbs as media in mindfulness and relaxation activities; use plants in decorations and other products and re-visited the mindfulness techniques, and promoted reflection on the participants’ experiences | Usual training in sheltered workshops or work placements during the study period, include work-related tasks (craft or manufacturing work), simulated work training, and coaching |
| Szczepańska-Gieracha et al. (2021) | General fitness training, psychoeducation and 8sessions of VRTierOne therapy | General fitness training, psychoeducation |
| Tao et al. (2020) | Perform the making of a flower basket | A computer task |
| Tu et al. (2020) | Grass Doll, Kokedama, Rocky Leaf Prints, and Herb Tasting and Smelling | Watching TV |
| Van Den Berg and Custers (2011) | Outdoor Gardening: light activities such as pruning of plants and bushes, weeding and the removal of dead flowers, sowing or planting on their own plots. Heavy activities, such as cutting of large branches or digging were not allowed. | Indoor Reading, read popular magazines that were screened for the absence of visual or verbal contents related to nature |
| Wei et al. (2020) | Sowing | - |
| Gong and Chen（2021） | Embossing, making sachets, drinking tea, arranging flowers, walking by the lake, and meditation in the forest | - |
| Chan et al. (2022) | Attended eight sessions of horticultural activities | Not participate in any horticultural activities and only participated in the routine activities arranged in their community centers |
| Curzio et al. (2022) | Horticultural therapy consisted of 24 sessions carried out twice a week for a total duration of 12 weeks (September–November 2020) | Nutritional counselling and rehabilitation, psychiatric, and psychopharmacological treatment as well as psychological intervention |
| Du et al. (2022) | The subjects selected flowers, trimmed them and inserted them into a vase with water. Then, the position of the flower branches was adjust appropriately until they are satisfied, and the flower arrangement is then complete. | - |
| S.-M. Lee et al. (2022) | Participated in the apartment community garden program focusing on horticultural education | Not participate in the program after the pre-test |
| Odeh et al. (2022) | Planting seeds (2 sessions) and vegetative propagation by cuttings/divisions (2 sessions); transplanting (2 sessions); and simulated harvest (2 sessions) | Art-making |
| Tao et al. (2022) | Excavation, transplanting and watering | Choose to read according to their preference or appreciate the surrounding environment freely to relax |

Setting

| **Studies** | **Settings** | **Types** | **Outdoor types** | **Design** |
| --- | --- | --- | --- | --- |
| A.-Y. Lee et al. (2018) | An occupational therapy room and rooftop garden of B Hospital | Indoor&Outdoor |  | Therapeutic Design |
| Chalmin-Pui et al. (2021) | Community garden | Outdoor | Garden | - |
| Chen et al. (2015) | Indoor classroom | Indoor |  | Non-Therapeutic Design |
| Dewi et al. (2017) | Garden and greenhouses at the Center for Field Science Research and Education (FSC) | Indoor&Outdoor |  |  |
| Gonzalez et al. (2011) | Farm settings | Outdoor | Farm |  |
| Han et al. (2018) | A farm (area: 991.7 m2) located in Suwon | Outdoor | Farm | Non-Therapeutic Design |
| Hassan et al. (2019) | A experimental room | Indoor |  | Non-Therapeutic Design |
| Hawkins et al. (2011) | a site consisting of many other gardening plots | Outdoor | Garden |  |
| Huang et al. (2017) | A Meeting Room（A session oudoor） | Indoor |  | Non-Therapeutic Design |
| Kam and Siu (2010) | The New Life Farm, in the five outdoor theme gardens of the Farm, namely the Sensory Garden, Activity Garden, Farm Garden, Display Garden and Practical Garden | Outdoor | Farm |  |
| Kim et al. (2021) | The experiment environment was created in a space(180 cm · 160 cm) at the campus of Konkuk University in Seoul, South Korea | Indoor |  | Therapeutic Design |
| M.J. Lee et al. (2018) | The Wee Class counseling office (W3.7 × L4.0 × H2.8 m3) | Indoor |  |  |
| M.-S. Lee et al. (2015) | A greenhouse room | Indoor |  |  |
| Meore et al. (2021) | A three-acre sustainable vegetable garden with indoor classrooms and a teaching greenhouse | Indoor&Outdoor |  |  |
| Pálsdóttir et al. (2013) | a specially designed health garden on the campus of the Swedish University of Agricultural Sciences in Alnarp, Sweden(two-hectare) | Outdoor | Garden |  |
| Park et al. (2017a) | A garden space (8*5 m) in the D senior community center | Outdoor | Garden |  |
| Park et al. (2017b) | A space (1.5 *1.7 m) with a table and chair in a laboratory at the Konkuk University campus | Indoor |  | Non-Therapeutic Design |
| Shao et al. (2020) | A quiet room | Indoor |  |  |
| Siu et al. (2020) | Not mentioned | Not mentioned |  |  |
| Szczepańska-Gieracha et al. (2021) | In the Virtual Therapeutic Garden | Virtual |  |  |
| Tao et al. (2020) | A laboratory room at the College of Landscape Architecture | Indoor |  |  |
| Tu et al. (2020) | 98 square meters room | Indoor |  |  |
| Van Den Berg and Custers (2011) | A garden space (8 × 5 m); 21 hectares containing 440 plots | Outdoor | Garden |  |
| Wei et al. (2020) | Beijing Jianwei Street warm home | Indoor |  | Non-Therapeutic Design |
| Gong and Chen（2021） | Jinan Forest Park | Outdoor | Park | Therapeutic Design |
| Chan et al. (2022) | Community centers | Indoor |  |  |
| Curzio et al. (2022) | The garden and/or in a PVC tunnel greenhouse of the clinical unit | Indoor&Outdoor |  |  |
| Du et al. (2022) | A quiet room | Indoor |  |  |
| S.-M. Lee et al. (2022) | The community garden | Outdoor | Garden | Non-Therapeutic Design |
| Odeh et al. (2022) | A therapeutic horticulture greenhouse | Indoor |  |  |
| Tao et al. (2022) | The campus of Zhejiang Normal University | Outdoor | Campus | Non-Therapeutic Design |

The performers

| **Studies** | **Performers** |
| --- | --- |
| A.-Y. Lee et al. (2018) | A horticultural therapist and 14 assistant horticultural therapists |
| Chalmin-Pui et al. (2021) | None |
| Chen et al. (2015) | A female horticultural therapist |
| Dewi et al. (2017) | Not mentioned |
| Gonzalez et al. (2011) | Not mentioned |
| Han et al. (2018) | A researcher (a master’s student in horticultural therapy) and three assistant therapists (also master’s students in horticultural therapy), with assistance from a social worker and staff member from the mental health centres in Suwon |
| Hassan et al. (2019) | None |
| Hawkins et al. (2011) | None |
| Huang et al. (2017) | A horticultural therapist |
| Kam and Siu (2010) | A registered occupational therapist |
| Kim et al. (2021) | None |
| M.J. Lee et al. (2018) | A professional therapist with a first-grade horticultural therapist certificate and an assistant counselor |
| M.-S. Lee et al. (2015) | None |
| Meore et al. (2021) | A registered horticultural therapist |
| Pálsdóttir et al. (2013) | An occupational therapist and a horticulturist |
| Park et al. (2017a) | An instructor |
| Park et al. (2017b) | None |
| Shao et al. (2020) | None |
| Siu et al. (2020) | Eight horticultural therapy participants,the researcher, a qualified counsellor, conducted the focus group and a research assistant |
| Szczepańska-Gieracha et al. (2021) | - |
| Tao et al. (2020) | None |
| Tu et al. (2020) | Not mentioned |
| Van Den Berg and Custers (2011) | An experimenter |
| Wei et al. (2020) | An experimenter |
| Gong and Chen（2021） | Not mentioned |
| Chan et al. (2022) | Not mentioned |
| Curzio et al. (2022) | Not mentioned |
| Du et al. (2022) | The researcher |
| S.-M. Lee et al. (2022) | An urban agricultural manager and an assistant |
| Odeh et al. (2022) | A horticulture master’s student, study coordinator, and trained study staff |
| Tao et al. (2022) | None |
